# Diversity of Neotropical stalked-puffball: Two new species of *Tulostoma* with reticulated spores

**DOI:** 10.1371/journal.pone.0294672

**Published:** 2023-12-13

**Authors:** Tiara Sousa Cabral, Bianca Denise Barbosa da Silva, Ruby Vargas-Isla, Jadson José Souza de Oliveira, Jorge Alves da Silva Ferreira, Laís Castro, María Paz Martín, Noemia Kazue Ishikawa

**Affiliations:** 1 Departamento de Parasitologia, Universidade Federal do Amazonas, Manaus, Amazonas, Brazil; 2 Departamento de Botânica, Instituto de Biologia, Universidade Federal da Bahia, Salvador, Bahia, Brazil; 3 Coordenação de Biodiversidade, Instituto Nacional de Pesquisas da Amazônia, Manaus, Amazonas, Brazil; 4 Programa de Pós-graduação em Botânica, Instituto Nacional de Pesquisas da Amazônia, Manaus, Amazonas, Brazil; 5 Bela Vista Ambientes, Paraty, Rio de Janeiro, Brazil; 6 Fazenda Bananal, Paraty, Rio de Janeiro, Brazil; 7 Departamento de Micología, Real Jardín Botánico-CSIC, Madrid, Spain; Friedrich Schiller University, GERMANY

## Abstract

Species of the genus *Tulostoma* are easily recognizable by the presence of a spore sac, with a mouth from which spores are released, attached to a stipe. *Tulostoma* is a species-diverse genus with a worldwide distribution, and some attempts were made to delimitate species and to evaluate reliable taxonomic-informative characteristics for species identification. However, there is a notable information gap regarding Neotropical species, especially for geographic distribution and DNA data, which hampers further understanding of the infrageneric diversity, evolution, and ecology of this genus. Based on morphological analysis, molecular phylogenetics and geographic distribution, we propose here two new species of *Tulostoma* with reticulated spores, from the two threatened Brazilian geographical areas, Atlantic Forest and “campos rupestres” (rupestrian grassland), as well as we provide notes on the taxonomic rank of *Tulostoma exasperatum* var. *ridleyi*.

## Introduction

Commonly known as stalked-puffballs, species of the genus *Tulostoma* Pers. are easily recognizable by the presence of a spore sac, with a mouth from which spores are released, attached to a stipe, which itself may be visible or may grow belowground [1]. This genus was first proposed by Persoon [2], with *Tulostoma brumale* Pers. as the type species; the name *Tulostoma* was later sanctioned by Persoon [3]. *Tulostoma* is a species-diverse genus with a worldwide distribution, which includes 276 legitimate species according to MycoBank [4].

In an attempt to delimitate species, Wright [5] defined the mouth and spores (size and ornamentation) as reliable primary characteristics. Wright [1] expanded these studies and published a classification based on macro- and micromorphological characters, incrementing Pouzar’s proposition of four sections: Brumalia Pouzar, Poculata Pouzar, Fimbriata Pouzar, and Volvulata Pouzar [6]. Wright’s classification proposed new subgenera, series, and sections, based mainly on the type of mouth and exoperidium [1].

With the application of molecular techniques to fungal systematics, some new attempts to delimitate species have been implemented. Studying European specimens, Jeppson et al. [7] reported high species diversity, including five new species to science. They also found that some of the characters commonly used to differentiate species are homoplasious or plesiomorphic but suggested that spore ornamentation and endo- and exoperidium characteristics may be good features to delimit species. In addition to the morphological aspects, internal transcribed spacer (ITS) regions between small-subunit and large-subunit ribosomal RNA (rRNA) genes have widely been used in DNA barcoding to identify *Tulostoma* species [8–11].

However, there is a notable information gap regarding Neotropical species, a fact that Wright himself perceived [1]. Despite the efforts of taxonomists in studying *Tulostoma* diversity in this important geographic area [9, 12–18], there is a paucity of geographic distribution data and especially of DNA data, which hampers further understanding of the infrageneric diversity, evolution, and ecology of this genus. For example, species with smooth spores tend to occur in dry areas, while species with ornamented spores occur in humid areas [7, 19, 20], but this has been tested to date only for Argentinian and European species.

Compared to other localities and taxa, there are few studies on *Tulostoma* species diversity in Brazil. Currently, 20 species are registered in Brazil from five of the six Brazilian biomes: Amazonia rainforest, Atlantic Forest, Caatinga, Cerrado, and Pampa [13–15, 18, 21, 22]. Brazil is a global biodiversity hotspot, harboring a vast array of fungal, plant, and animal species found nowhere else on Earth. Therefore, it has a high potential for species discovery, as is probably the case for *Tulostoma*. The most recent new species for the country were *Tulostoma irregulireticulatum* Dourado-Barbosa, R.L. Oliveira, A.A. Lima, Baseia & R. Cruz [22], *T*. *catimbauense* A.A. Lima, Accioly, Baseia & M.P. Martín and *T*. *deltaconcavum* A.A. Lima, Accioly, Baseia & M.P. Martín [18].

From the Brazilian species, *Tulostoma exasperatum* Mont. is so far the most represented species. It was first described from Cuba and is shown to be easily distinguished by the spiny exoperidium, scaly stipe, and reticulated spores, and is one of the few species with lignicolous habitat [1]. *Tulostoma ridleyi* Massee, described in 1899 from Malaysia is another very similar species, which differs mainly in the basidioma size and the sizes and shapes of the spores and endoperidial verruca [1]. Therefore, Wright [1] changed the species status to the variety *T*. *exasperatum* var. *ridleyi* (Massee) J.E. Wrigth. After the description of the species, several specimens of *T*. *exasperatum* were recorded, and it is now known to be widely distributed in the African, Asian, and American tropics [23–29]. On the other hand, the few records of *T*. *exasperatum* var. *ridleyi* from Malaysia, Singapore, and Thailand [30] suggest that this taxon is endemic to Southeast Asia, but further analyses are needed to elucidate the taxonomic rank. Similarly, because several widely distributed mushroom species may in fact be different evolutionary units [31–34], studies on the diversity of *T*. *exasperatum* sensu Wright [1] are also needed.

Here, we propose two new species of *Tulostoma* with reticulated spores found in Brazil, closely related to *T*. *exasperatum*, but from different phytophysiognomies and different substrates (wood and soil), based on morphology and DNA phylogenetic data. In addition, we provide notes on the taxonomic rank of *T*. *exasperatum* var. *ridleyi*.

## Materials and methods

### Sampling and morphological analysis

**Fig 1:**
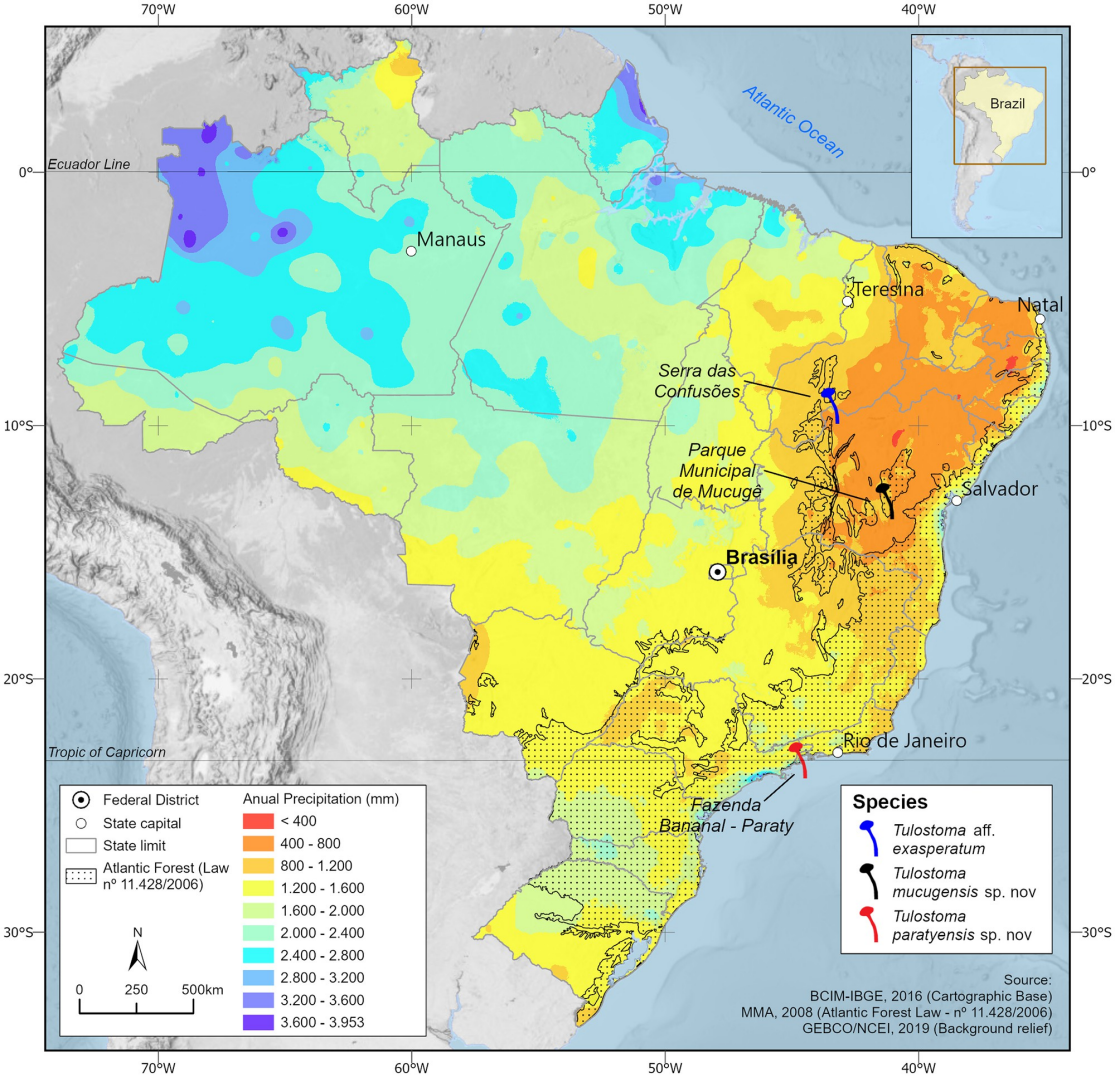
Geographical distribution of Neotropical species studied here. Map: Clayton Bit-tencourt Junior. The map image is from GEBCO (General Bathymetric Chart of the Oceans) Gridded Bathymetric Data and it is in the public domain.

Specimens were collected during field trips to areas of the Atlantic Forest (AF) in southeast Brazil (Paraty, state of Rio de Janeiro–RJ–three specimens collected) and rocky fields (rupestrian grassland, “campos rupestres”) of northeast (Mucugê, in Chapada Diamantina range, state of Bahia–BA–a single specimen collected) Brazil (Fig 1). The AF is considered a biodiversity hotspot, found only in Brazil, with high levels of endemicity, although it has suffered severe habitat fragmentation and other threats [35, 36]. The sample area in RJ was Fazenda Bananal, a private reserve for ecotourism and nature education, which includes part of the AF. It is a coastal area with a temperate-dry winter with hot summer (“Cwa” in Köppen’s climate classification), at sea level, with a mean annual rainfall of 3407 mm and mean temperature of 21°C. The “campos rupestres” are characterized by herbaceous and bushy vegetation associated with rocky outcrops and sandy soil on the tops of mountains (above 900 m), with recognizable levels of endemicity [37]; the climate is semihumid (“Aw” in Köppen’s climate classification], with mean annual rainfall of 1100 mm and mean temperature of 20°C [38]. We followed Vargas-Isla et al. [39] for collection, storage, and herborization of specimens. This study is registered at SisGen (Sistema Nacional de Gestão do Patrimônio Genético e do Conhecimento Tradicional Associado) under ID A6DBD2C; collections made at Fazenda Bananal were authorized in writing by the managers and specimens were transported under SISBIO 85230–1 permission. Collections made at Parque Nacional de Mucugê does not require permissions.

Morphological descriptions were obtained based on both fresh and dried basidiomata using an M205 C stereo microscope coupled with an MC 190 camera, and a DM 2500 optical microscope (Leica, Wetzlar, Germany). Colors were defined based on Küppers [40] and mycelial cord/rhizomorph descriptions were based on Yafetto [41]. For micromorphological characters, freehand sections of different structures (peridium, stipe, rhizomorph, spores) were mounted in 5% KOH or distilled water, and stained with cotton blue, Congo Red or Melzer’s reagent, and observed under a light microscope. Thirty spores were measured, including the ornamentation, where n = number of randomly measured basidiospores, x = mean ± standard deviation of basidiospore diameter and height (including ornamentation), and Qm = mean height/width quotient. We followed the spore shape definitions of Bas et al. [42]. Scanning electron microscopy (SEM) was performed at Centro Multiusuário para Análise de Fenômenos Biomédicos (CMABio) of Universidade Estadual do Amazonas (UEA) and Laboratório de Microscopia Eletrônica (LAMUME—Institute of Physics) of Universidade Federal da Bahia, following previously described methods [43]. Additional figures of macro and microcharacters can be found at Cabral et al. [44].

### Phylogenetic analyses and species delimitation approach

To increase the geographic coverage of the sampling, one specimen registered as *T*. *exasperatum* was loaned from UFRN-Fungos herbarium and included in phylogenetic analyses, as well as a specimen from Singapore deposited at MA herbarium (MA-Fungi 83796). DNA was extracted from dried material using a DNeasy Plant Mini kit (Qiagen, Hilden, Germany). Three DNA regions were obtained the internal transcribed spacer (ITS), the large subunit of the nuc-RNA gene (nucLSU), and translation elongation factor subunit 1 alpha (TEF1a), using primers pairs ITS1-ITS4 [45], LR0R-LR5 [46], and EF983F-F2218R [47], respectively, for both amplification and sequencing. Sequences generated in this study were visualized and assembled using Geneious R6.1 (Biomatters Ltd., Newton, New Zealand), and submitted to a BLAST search. The newly generated sequences were aligned to homologous sequences available in GenBank, mostly from Jeppson et al. [7] (S1 Table), and edited manually using the MUSCLE alignment tool in AliView v 1.26 [48]. Seventy-nine taxa were included in phylogenetic analysis, with *Calvatia caatinguensis* (UFRN-Fungos 2945) and *Lycoperdon subperlatum* (KA12-0981) forming the outgroup, following previous publications [7, 29]. Each DNA region was aligned separately and the substitution model was chosen for each matrix using MrModelTest [49]. Each matrix was composed of all specimens, and for the specimens lacking a DNA region, it was treated as missing data. Then the three matrices were concatenated into a single matrix, which was subjected to Bayesian phylogenetic analysis (BA) with MrBayes [50]. This consisted of two parallel runs executed with four incrementally heated simultaneous MCMC simulations over 10 million generations, with trees sampled every 1000 generations, with the first 25% discarded at the tree summarizing stage. Maximum likelihood (ML) analysis was run in RAxML [51] using the same partitioned dataset, with estimated proportion of invariable sites (GTRGAMMA + I). Support values are given as posterior probabilities (PP) for BA analysis and bootstrap (BP) for ML on the nodes of the tree. The substitution model and phylogenetic analyses were implemented in CIPRES Science Gateway [52]. Trees were visualized and edited in FigTree version 1.4.2 (http://tree.bio.ed.ac.uk/software/figtree/). All data are available in TreeBASE under submission ID 30727.

The Bayesian Posterior Tree Poisson (bPTP) analysis was applied for species delimitation [53]. The bPTP analysis was run in a web server (http://species.h-its.org/ptp/) with 500,000 MCMC generations and the remaining default settings, using the same Bayesian tree obtained in previous analysis. We also generated distances matrix based on ITS sequences, calculated between one representative of each species defined by bPTP, using dist.dna function of the ape package [54] with “raw” model, and MEGA5 software to calculate ingroup mean distances [55]; the distances were plotted in a heat map using heatmapSpp function of spider package [56] for R environment.

#### Nomenclature

The electronic version of this article in Portable Document Format (PDF) in a work with an ISSN or ISBN will represent a published work according to the International Code of Nomenclature for algae, fungi, and plants, and hence the new names contained in the electronic publication of a PLOS article are effectively published under that Code from the electronic edition alone, so there is no longer any need to provide printed copies.

In addition, new names contained in this work have been submitted to MycoBank from where they will be made available to the Global Names Index. The unique MycoBank number can be resolved and the associated information viewed through any standard web browser by appending the MycoBank number contained in this publication to the prefix http://www.mycobank.org/MB/. The online version of this work is archived and available from the following digital repository: PubMed Central, LOCKSS.

## Results

### Phylogenetic analyses

**Fig 2:**
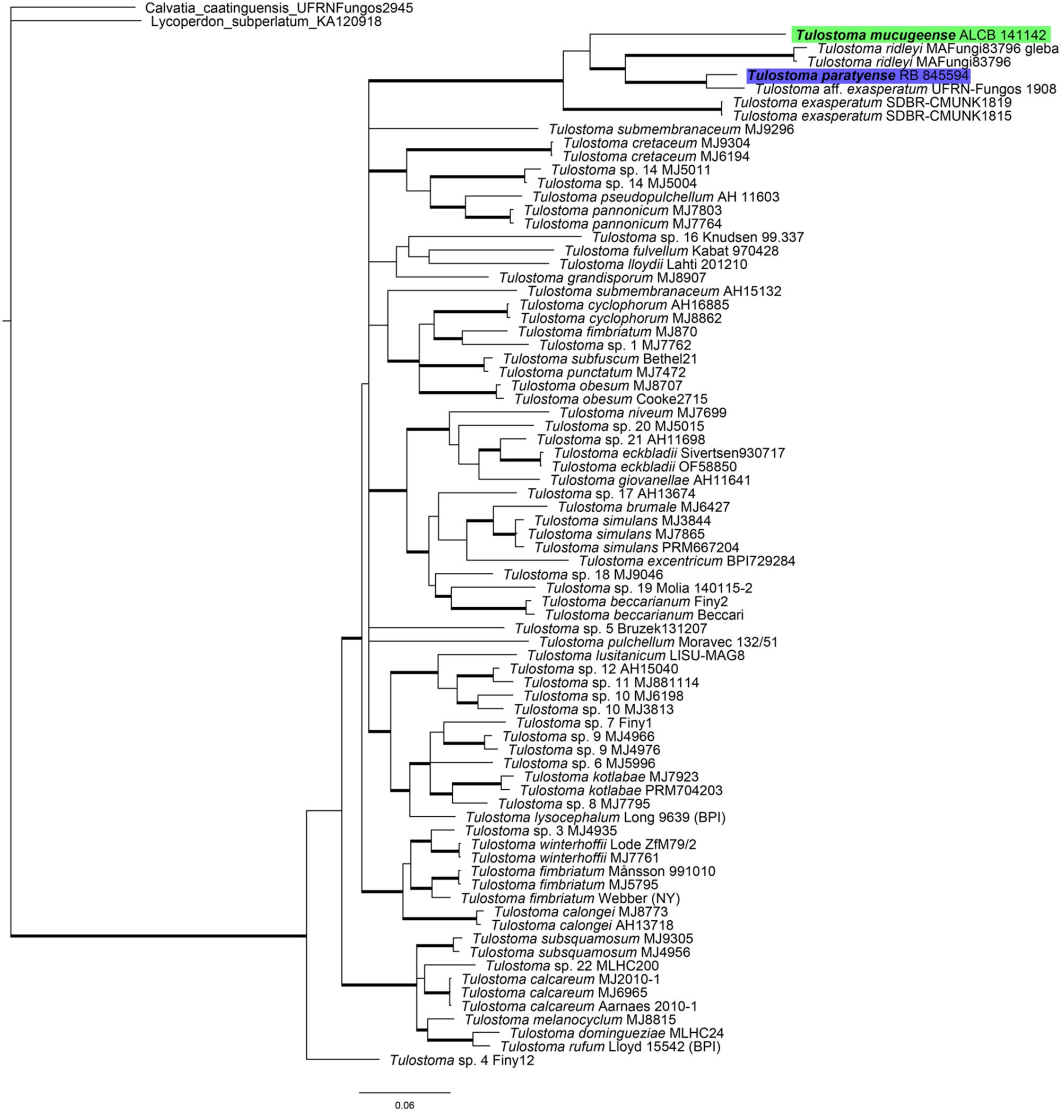
Consensus phylogenetic tree of genus *Tulostoma*, obtained by Bayesian inference with ITS, nucLSU and Tef1-α. Colors indicate the new species proposed here: *T*. *mucugeense* in green and *T*. *paratyense* in blue. Thicker branches indicate posterior probabilities values ≥ 95%.

Nine new sequences from the analyzed *Tulostoma* were generated in this study (5 ITS, 3 nucLSU, and 1 TEF1a). The concatenated matrix consisted of 77 sequences of ITS, 64 nucLSU, and 24 TEF1a, with 2813 characters, of which 794 were from ITS, 886 were from nucLSU, and 1133 were from TEF1a. For the three alignments (ITS, nucLSU, and TEF1a), the GTR+I+G model was selected using MrModelTest. The resulted phylogenetic tree of both methods differ mainly in the most internal nodes, where Bayesian analysis resulted in an unresolved tree topology. On the other hand, Maximum Likelihood analysis resulted in a topology with better resolution, but with low support values mostly on internal nodes. However, the species clades were congruent in both analyses (ML phylogenetic tree can be found in S1 Fig). In the phylogenetic tree (Fig 2 and S1 Fig), most clades of the European species were consistent with Jeppson et al. [7]. However, one clade grouped the Neotropical and three South Asian specimen, all of which had reticulated spores and verrucose or spiny exoperidium (similar to *T*. *exasperatum*), with high a support value (PP = 1, BP = 100). Within the new clade with reticulated spores, we delimited five species-level clades, two of which are described here as new species and one that has a change in the status rank, as discussed in the following sections.

The bPTP method estimated a mean number of 59 partitions assigned to putative species by simple heuristic search (bayesian) and 58 by maximum likelihood. In both searches, the clade with reticulated-spores returned 5 species with high support values: *T*. *mucugeense* (1.00), *T*. *ridleyi* (0.87), *T*. aff. *exasperatum* from Brazil (1.00)., *T*. *exasperatum* from Thailand (0.99) and *T*. *paratyense* (1.00) (see S2 Data; the tree topologies are available at Cabral et al. [44]). Additionally, the genetic p-distances values ranged between 0% to 16%, with the intraspecific mean distances of 5.8%; interestingly, the mean distances between reticulated-spores group and other *Tulostoma* species is of 10%. Both the genetic distances matrix and a heat map can be found at S1 Data and S2 Fig.

### Taxonomy

**Fig 3:**
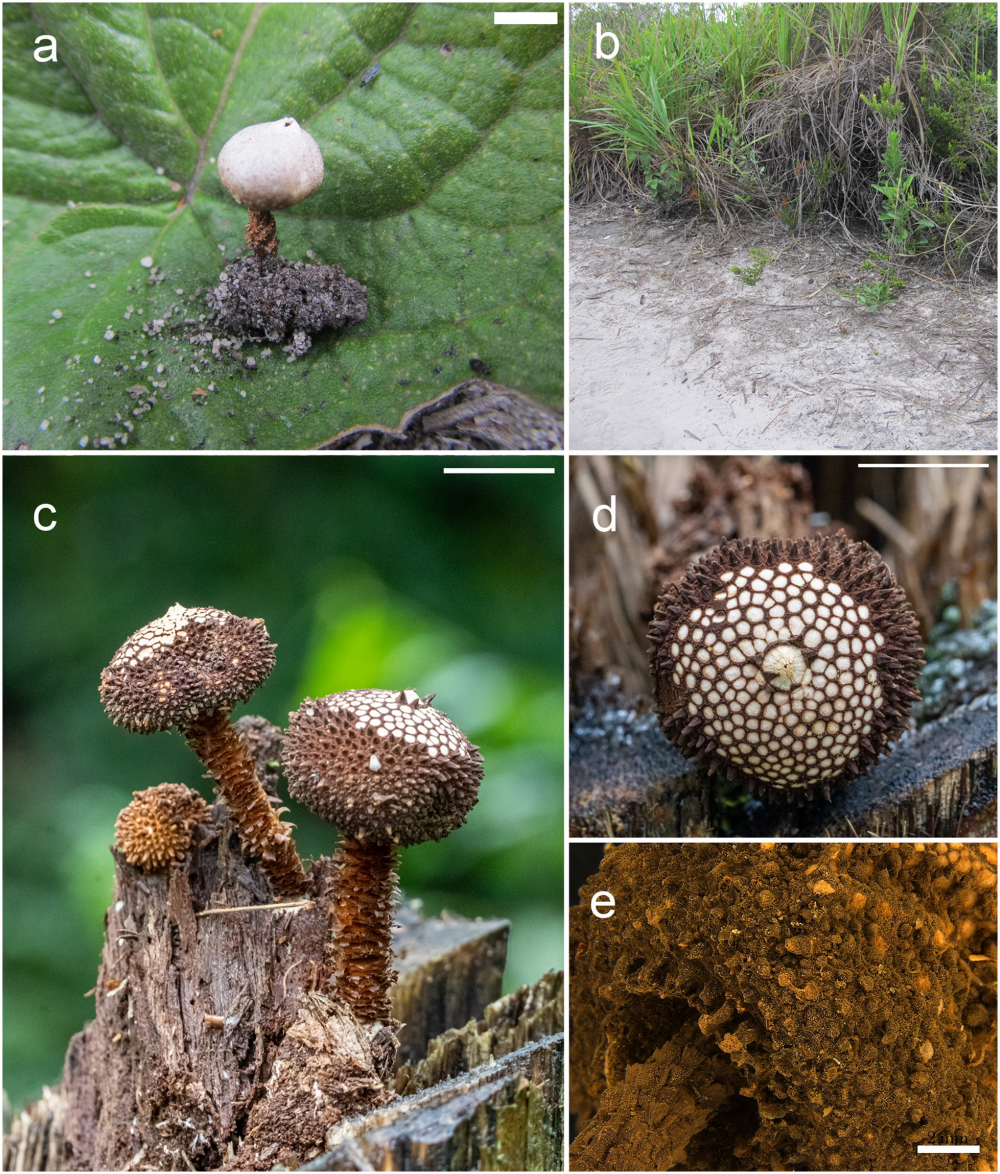
*Tulostoma mucugeense*: a. macromorphology (bar = 5 mm), b. habitat type. *Tulostoma paratyense*: c. macromorphology and gregarious habit (bar = 10 mm), d. mouth and exoperidium (bar = 10 mm), e. socket (bar = 2 mm). Photos (c, d): Rafael E. de Freitas.

**Fig 4:**
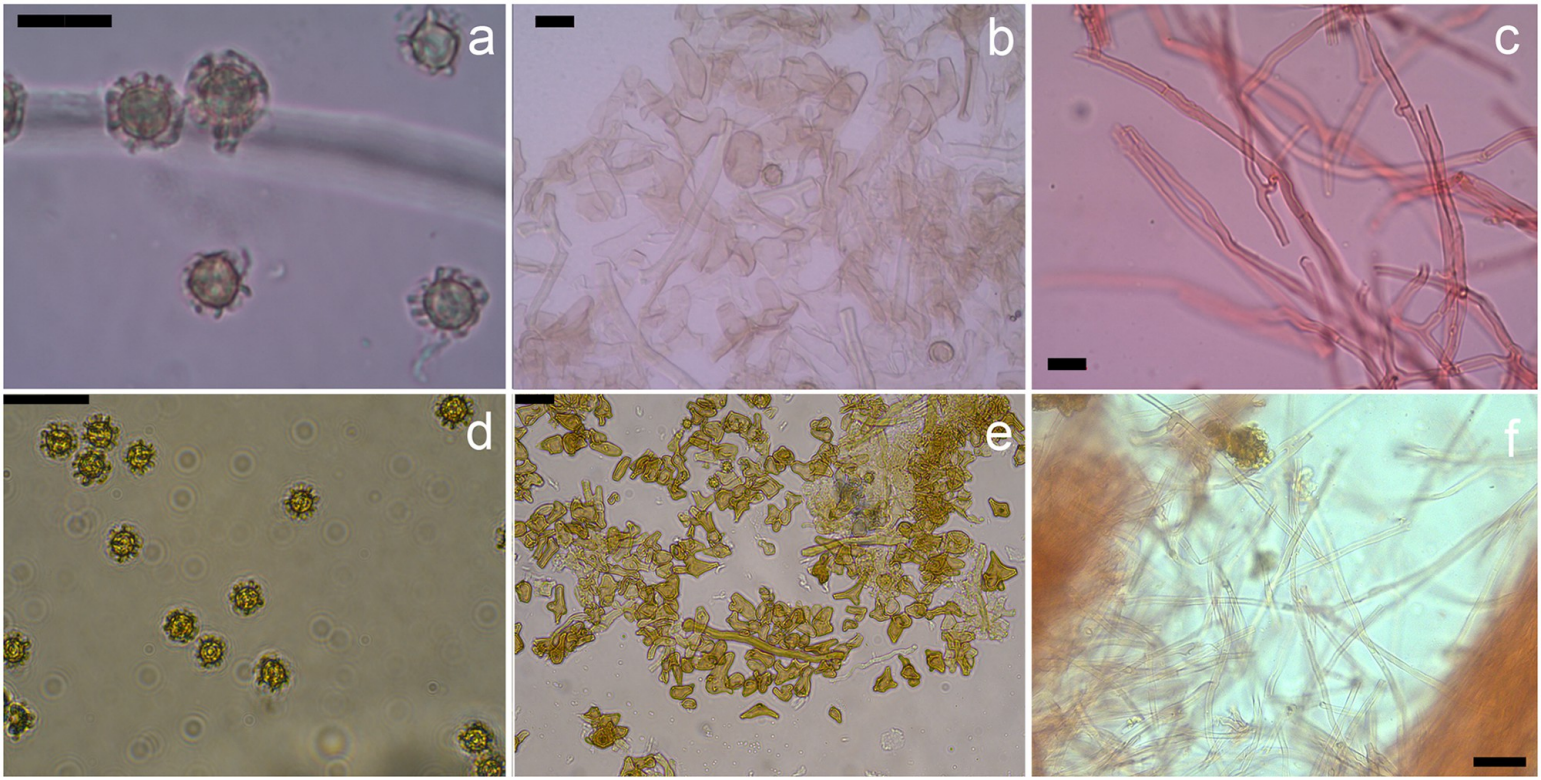
Micromorphology: a, d. spores (bars: a = 5 μm, d = 10 μm); b, e. hyphae of exoperidium (bars = 10 μm); c, f. hyphae of mycelial base (bars: c = 10 μm, f = 20 μm). a, b, c. *Tulostoma mucugeense*; d, e, f. *Tulostoma paratyense*.

**Fig 5:**
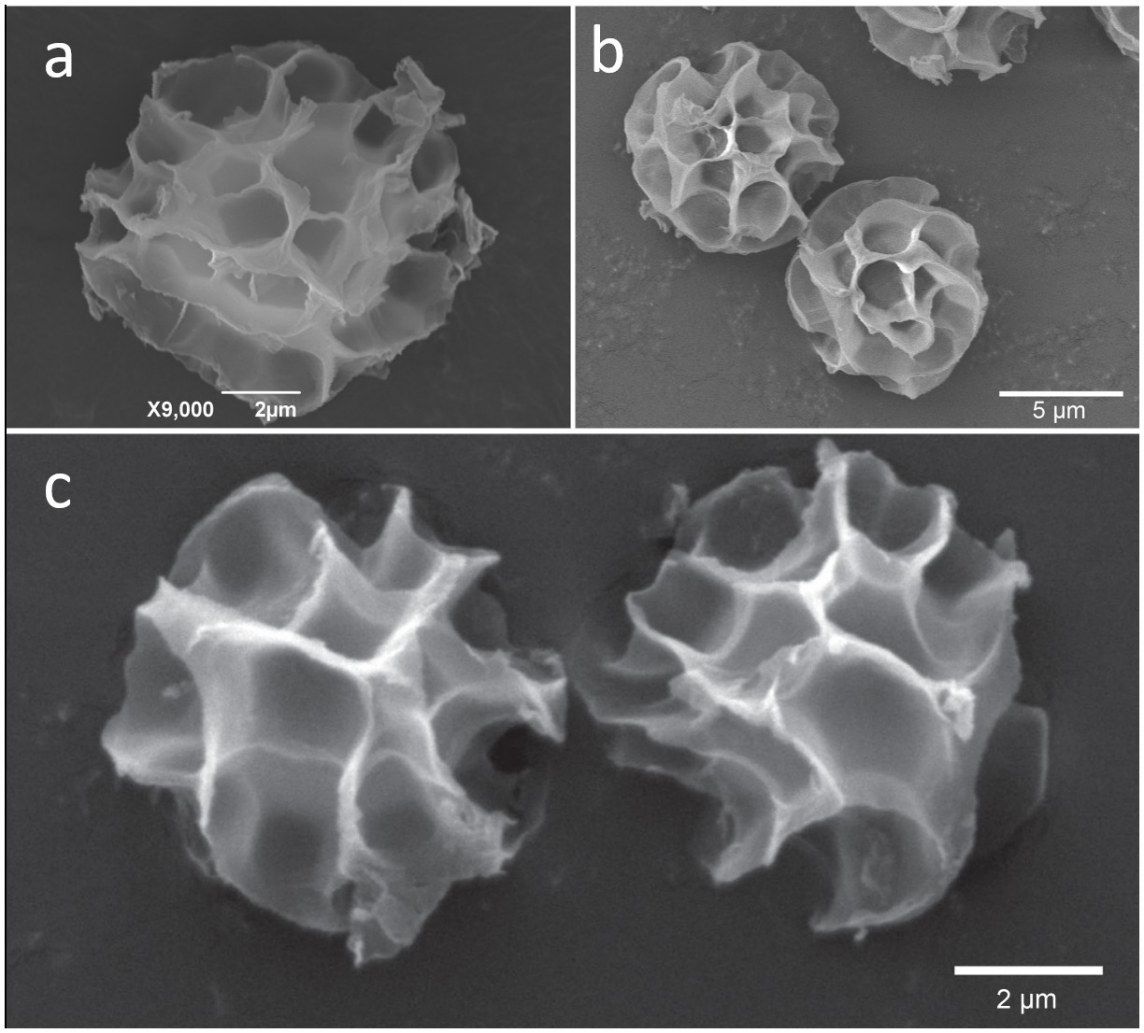
Spores’ morphology under SEM. a. *Tulostoma mucugeense*; b. *Tulostoma paratyense*; c. *Tulostoma* aff. *exasperatum* UFRN-Fungos 1908.

***Tulostoma mucugeense*** B.D.B. Silva & T.S. Cabral, sp. nov., Figs 3a, 3b, 4a–4c and 5a.

Mycobank: MB847695 [urn:lsid:mycobank.org: 847695]

Diagnosis: The species is characterized by spores with halos under light microscopy, helicoid with uneven alveoli under SEM, measuring 8 × 10 μm; fibrillose mouth, exoperidium verrucose, and found growing on sandy soil.

Holotype: Brazil, Bahia, Mucugê, Parque Nacional de Mucugê, -12.983333, -41.333056, 05 Apr. 2018, leg. Silva, B.D.B. et al. (ALCB 141142!, ITS, nucLSU Genbank sequences: OQ626704, OQ626710).

Etymology: In reference to the type locality, Mucugê (Bahia, Brazil).

Macromorphology: Spore sac subglobose, up to 8 mm high × 5 mm wide. Exoperidium verrucose, brown (N_70_Y_90_M_50_) with areolate surface, granulose-membranous on the lower third and persisting in parts of the rest, falling with maturity first at the apex and later at the base, leaving scars on the exoperidium. Endoperidium smooth, light brown (A_70_M_20_C_10_). Mouth fibrillose, concolor with the endoperidium. Socket slightly detached from the stipe, formed mainly by the pyramidal spines. Stipe short and slender, 10 mm in high × 3 mm wide, surface scaly, with longitudinal scales densely distributed, light brown (Y_80_M_50_C_30_), basally ending in a small bulb. Gleba beige to light brown (Y_80_M_40_C_30_) when mature, pulverulent.

Micromorphology: Basidiospores globose, ornamented, 8 × 10 μm (x = 8.8 ± 0.78 × 8.8 ± 0.8, Qm = 1, n = 30) including ornamentation, brown in 5% KOH, spiny under light microscopy, helicoid with uneven alveoli under SEM. Capillitium 3–5 μm, thick-walled, septate, lumen visible, hyaline in 5% KOH. Exoperidium formed by hyphae of variable shape, yellowish, 4–8 μm. Endoperidium formed by long hyphae, hyaline to yellowish, thick (2–4 μm), with widened septa. Stipe context formed by thin-walled hyphae, hyaline in 5% KOH, septate, with clamp connections, 2–4 μm wide. Scales of stipe formed by parallel organized hyphae, septate, light brown, 5–7 μm wide, brittle, thick-walled. Mycelial base formed by parallel organized hyphae, thin-walled, septate, 3–4 μm wide, thick-walled hyaline to light brown in 5% KOH.

Habitat and distribution: Found growing on sandy soil, known from highlands of the state of Bahia (“campos rupestres”).

Notes: *Tulostoma mucugeense* is a species with reticulated spores and a fimbriated mouth. It differs from *Tulostoma paratyense* n. sp., *T*. *exasperatum*, and *T*. *ridleyi* by its spiny exoperidium, from *T*. *opacum* Long and *T*. *rickii* Lloyd by its membranous exoperidium, and from *T*. *portoricense* J.E. Wright by its hyphal exoperidium. In addition to presenting reticulated spores and a fimbriated mouth, *T*. *transvaalii* Lloyd also has exoperidium verrucose as in *T*. *mucugeense*. However, it differs due to the larger spores in the latter (4.6–5.7 μm). *Tulostoma exasperatosporum* J.E. Wrigth also has reticulated spores but has a tubular mouth and membranous exoperidium [1].

***Tulostoma paratyense*** T.S. Cabral & B.D.B. Silva, sp. nov., Figs 3c–3e, 4d–4f and 5b.

Mycobank: MB847959 [urn:lsid:mycobank.org: 847959]

Diagnosis: The species is characterized by spores with a halo under light microscopy, helicoid with uneven alveoli under SEM, measuring 7.5–8.2 × 10.3–10.6 μm; fibrillose-fimbriate mouth; exoperidium spiny and lignicolous habitat.

Type: Brazil, Rio de Janeiro, Paraty: Fazenda Bananal, -23.215611, -44.763167, 13 Dec. 2022, leg. Ferreira, J., Oliveira, J.J.S & Freitas, R.E..—JO2162 (Holotype: RB 845594!, ITS, nucLSU and TEF1a Genbank sequences: OQ626707, OQ626712, OQ658839).

Etymology: In reference to the type locality, Paraty (Rio de Janeiro, Brazil).

Macromorphology: Spore sac subglobose, up to 14 mm high × 21 mm wide. Exoperidium spiny, brown (N_80_A_60_M_70_) with areolate surface (mud-cracked), and presence of pyramidal spines up to 1.6 mm, each spine with a rounded ring at the base, more abundant closer to the mouth, falling down with maturity first at the apex and later at the base, leaving scars on the exoperidium. Endoperidium smooth, light brown (A_70_M_20_C_10_). Mouth fibrillose-fimbriate, concolor with endoperidium, but darker when mature. Socket slightly detached from the stipe, formed mainly by the pyramidal spines. Stipe long, 31–53 mm high × 3.5–4.5 mm wide, surface scaly, with longitudinal scales densely distributed, brown (N_80_A_60_M_70_), ending up in a cream yellow (N_10_A_50_M_20_) mycelium base. Gleba beige to light brown (N_40_A_70_M_50_) when mature, pulverulent.

Micromorphology: Basidiospores subglobose, ornamented, 7.5–8.2 × 10.3–10.6 μm (x = 9 ± 0.8 × 9.5 ± 0.6, Qm = 1,06, n = 30) including ornamentation, brown in 5% KOH, spores with a halo under light microscopy, helicoid with uneven alveoli under SEM. Capillitium 3–8 μm, thick-walled, septate, lumen visible, hyaline in 5% KOH. Presence of ribbon-like hyphae on gleba. Exoperidium formed by hyphae of variable shape, golden yellow in 5% KOH, same found on spines. Endoperidium formed by long hyphae, hyaline to yellowish, thick (2.5–6.5), with widened septa. Stipe context formed by thin-walled hyphae, hyaline in 5% KOH, septate, with clamp connections, 2.5–6.5 μm wide. Scales of stipe formed by parallel hyphae, septate, light brown, 3.5–7.9 μm wide, sometimes ramified, thick-walled. Mycelial base formed by hyphae thin-walled, septate, 2–4 μm wide, hyaline in 5% KOH.

Habitat and distribution: Found growing on wood (“Pau-jacaré”, *Piptadenia gonoacantha* (Mart.) J.F.Macbr.), gregarious; known so far from AF in Rio de Janeiro.

Notes: *Tulostoma paratyense* is morphologically very close to *T*. *exasperatum* but differs mainly in spore size and shape, growth habit, and size of scales on the stipe. In *T*. *exasperatum*, spores are globose and are up to 8 μm in length including ornamentation, under light microscopy with long spines projecting from the angles of the reticulum, which is not deciduous, may appear finger-like; cespitose growth habit [1], while in *T*. *paratyense* spores are subglobose measuring 7.5–8.2 × 10.3–10.6 μm, under light microscopy appearing spiny with a halo. *Tutolostoma paratyense* has larger and densely distributed scales on the stipe and gregarious growth habit, while in *T*. *exasperatum* the scales are smaller and the growth habitat is cespitose. *Tulostoma exasperatum* from Thailand [29] also has smaller basidiospores when compared to *T*. *paratyense*, and the spore ornamentation is more similar to *T*. *paratyense* than to *T*. *exasperatum* from Cuba [1]. *Tulostoma ridleyi* is also close morphologically but differs in spore shape and size (ovoid and up to 5–8 μm, respectively), larger stipe (120 mm), and the larger and more sparsely distributed spines on the exoperidium [1]. *Tulostoma opacum* and *T*. *rickii* also have a fimbriate mouth and reticulated spores; however, neither species presents a spiny exoperidium or a scaly stipe, and spores are strongly reticulated in *T*. *opacum* and apparently spiny under light microscopy in *T*. *rickii* [1]. *Tulostoma exasperatosporum* is another species with reticulated spores, but it has a tubular mouth, a lateritic exoperidium, and smaller spores [1] compared to *T*. *paratyense*. *Tulostoma squamosum* (J.F. Gmel.) Pers. has a stipe with dark brown scales but the scales have a scattered distribution throughout the stipe, mouth is tubular, and the spores are smaller and echinulate [10].

***Tulostoma ridleyi*** Massee, Bull. Misc. Inf., Kew (nos 153–154): 173 (1899)

= *Tulostoma exasperatum* var. *ridleyi* (Massee) J.E. Wright, Biblthca Mycol. 113: 99 (1987)

Description: Wright [1] and Calonge et al. [57].

Habitat and distribution: Lignicolous habit, distributed in Southeast Asia.

Material examined: Singapore, Chek Jawa Coastal Hill, 6 Feb. 2012, leg. J. Lai, Sing 2012–45 (MA-Fungi 83796).

Notes: According to Wright [1], *T*. *ridleyi* is morphologically close to *T*. *exasperatum* but has a larger basidioma and stipe, larger spines on the exoperidium, and larger spores. It also differs from *T*. *paratyense* in the slightly smaller spores, larger basidiome, larger spines on the exoperidium, and the cespitose growth habit. *Tulostoma mucugeense* is different from *T*. *ridleyi* mainly in the verrucose exoperidium. We also noted that the ornamentation of the spores is different under SEM; in *T*. *ridleyi*, the walls of alveoli are thicker and they are continuous throughout the spores, while in *T*. *exasperatum* these walls are thin and uninterrupted, making the ornamentation look like spines under light microscopy.

## Discussion

*Tulostoma* is a genus with a worldwide distribution but with little research of its neotropical diversity, especially including DNA data. Here, we focused on species from the Brazilian AF and rocky fields (“campos rupestres”), with reticulated spores, to evaluate their identity based on geographical distribution, phylogenetic position, and morphological characters. We identified two new species: *T*. *mucugeense*, characterized mainly by the verrucose exoperidium, reticulated spores, and fibrillose mouth in specimens from rocky fields (“campos rupestres”); and *T*. *paratyense*, characterized by the spiny exoperidium, reticulated spores, and fibrillose-fimbriate mouth in specimens from the AF. Although it is preferable to include multiple collections when describing a new fungal species, in some cases this is not possible (e.g., rare taxa, ephemeral basidiomata, etc.). For these cases, it is recommended that the species is described based on multiple line of evidence [58]. Although we have a single collection of *T*. *mucugeense*, we do provide macro and micro morphological and molecular data that supports the decision of describing it as a new species. Additionally, there are several examples of new fungal species being described based on single or a few collections. *Tulostoma portoricense* was described based on two collections, while *T*. *irregulireticulatum* was described using a single collection [12, 22]. *T*. *loonbanglaense* was described based on two collections, but both from exactly the same location [11].

Our phylogenetic analyses included six specimens with reticulated spores, where one specimen had a verrucose exoperidium, and five specimens were morphologically close to *T*. *exasperatum*—two from the Neotropics (UFRN-Fungos 1908 and RB 845594—Brazil) and three from Southeast Asia (Singapore, MA-Fungi 83796; and Thailand, SDBR-CMUNK1815 and SDBR-CMUNK1819). The specimen MA-Fungi 83796 included in our phylogenetic analyses was identified by Calonge et al. [57] as *T*. *exasperatum*. However, based on its geographic locality (Singapore), which is close to the type locality of *T*. *ridleyi* (Perak, Malaysia) [59], and based on illustrations and morphological description, we believe this specimen is in fact *T*. *ridleyi* described by Massee; therefore, we propose to preserve the species status of this taxon. The results of phylogenetic analysis supported this decision, as the species groups in a clade distinct from other reticulated spore species, with high support values (*T*. *paratyense*, *T*. aff. *exasperatum* UFRN-Fungos 1908, *T*. *exasperatum* from Thailand, and *T*. *mucugeense*; PP = 1, BP = 100), which have other differences (see Taxonomy section). In addition, a search for *T*. *exasperatum* at the Global Biodiversity Information Facility (GBIF) [60] and iNaturalist (https://www.inaturalist.org) limited to Southeast Asia returned five observations with associated photos, from which we observed a pattern of macrocharacters including large spines on the exoperidium and a cespitose growth habit. However, a detailed morphological analysis of microcharacters should be performed, especially for spore characterization. As suggested by Wright [1], we also believe this species may be endemic to this area, and it is important to include the type specimen in future phylogenetic analyses.

Specimens morphologically close to *T*. *exasperatum* grouped together in a clade with high support values (*T*. *paratyense* and *T*. aff. *exasperatum* UFRN-Fungos 1908, PP = 1, BP = 100). However, the specimens are morphologically different from each other. *Tulostoma* aff. *exasperatum* (UFRN-Fungos 1908) has smaller spores (up to 6.3 μm) with spiny ornamentation on light microscopy, the walls of alveoli on the spores are not continuous under SEM (Fig 5c), and the basidiomata have a cespitose growth habit [61]. For *T*. *paratyense*, the spores are up to 10 μm and have a halo formed mainly by the continuous walls of alveoli, and the basidiomata have a gregarious growth habit. *Tulostoma* aff. *exasperatum* (UFRN-Fungos 1908) is morphologically close to *T*. *exasperatum*, mostly in spore size and shape (spiny in light microscopy) and cespitose growth habit, but other specimens from this locality (Serra das Confusões, Piauí, Brazil) must be analyzed to determine the affinity to *T*. *exasperatum*.

*Tulostoma exasperatum* was originally described from Cuba. There are 89 records with this name in the GBIF database, which are from Africa (n = 6), Asia (n = 6), North America (n = 10), South America (n = 65), and two are not assigned to a continent. However, it is recognized that few fungal species are truly cosmopolitan, suggesting some level of endemism at regional scales [62]. Several examples have been published, and *T*. *exasperatum* may be such a case of a species complex and/or cryptic speciation, as we found morphological and phylogenetic differences between at least four *T*. *exasperatum*-like specimens, sufficient to delimit and define two species (*T*. *paratyense* and *T*. *ridleyi*). Additionally, specimens from Thailand included in analyses grouped in a different clade within the reticulated-spore group, with high support values (PP = 1, BP = 100). Although it was identified by the authors as *T*. *exasperatum* [29], our analyses show that it may be a putative species, since it is phylogenetically quite different from other *T*. *exasperatum*-like specimens (genetic distances between 8% to 10% based on ITS sequences). This is another reason to understand this species as a species complex or a case of cryptic speciation. Jeppson et al. [7] found a similar situation when studying European species, finding possible evidence of cryptic speciation and species complexes in *T*. *fimbriatum* Fr. and *T*. *cretaceum* Long, with strong geographical signals. Only a few descriptions of *T*. *exasperatum* from different localities are publicly available, especially from the type locality. The size and shape of spores, the presence or absence of the halo, and the size and distribution of the scales on the stipe may differentiate species similar to *T*. *exasperatum*.

*Tulostoma mucugeense* was collected in a rocky field from Chapada Diamantina, which is part of the Espinhaço Range in Brazil, the largest area of rupestrian grasslands, and an area considered to be an old, climatically buffered, and infertile landscape (OCBIL) [63]. The particularities of these areas favored the generation and maintenance of species endemism and microendemism, as observed for plants, amphibians, and birds [64–66]. These environments act simultaneously as ancient refugia and areas of recent speciation, which explains the observed high levels of diversity and endemism [63]. The plant diversity is mainly driven by the altitudinal and latitudinal range, the influence of the adjacent biomes (Cerrado, Caatinga, and the AF), habitat heterogeneity, and isolation between vegetation islands [63]. However, studies on fungal diversity have only recently been performed in Brazilian rupestrian grasslands, with the discovery of rare and new species [67–70]—and here we now include *T*. *mucugeense*—suggesting that these areas may also be species-diverse for fungal taxa. Nonetheless, further studies are necessary to assess this diversity and to understand the factors driving fungal diversity in rupestrian grasslands.

The rupestrian grasslands and AF have tropical semihumid and humid climates, respectively, and both new species found in these environments have reticulated spores. Although there is a tendency for species to develop smooth spores in dry areas and reticulated spores in humid areas, we show here that there may be species with reticulated spores in semihumid climates. In addition, at least two species with smooth spores have been reported from humid areas of Brazil, *T*. *cretaceum* Long and *Tulostoma obesum* Cooke & Ellis, both recorded from the AF in northeast Brazil [14]. Further studies are needed to test the hypothesis that species with ornamented spores are likely to occur only in humid areas, at least when extended to other neotropical areas.

Moreover, the two collection areas are known as biodiversity hotspots. The AF has been subject to severe threats, which have mainly caused habitat fragmentation; similarly, rupestrian grasslands are considered to be naturally vulnerable to disturbance, and would be severely affected by climate change [36, 65]. The finding of new species, especially understudied species, in these areas highlights the importance of planning and implementing conservation strategies. A good example is the “Fundação SOS Mata Atlântica”, a Brazilian environmental nongovernmental organization that has been monitoring and helping forest restoration in more than 500 parks and reserve forests [71]. Similar to SOS Mata Atlântica initiative, the Parque Municipal de Mucugê is a conservation area that aims to preserve ecosystems, promotes sustainable activities through environmental education and sustainable tourism. *Tulostoma mucugeense* was collected from Parque Municipal de Mucugê, and *T*. *paratyense* was collected from Fazenda Bananal, a reference company in the Atlantic Forest for it combines forest restoration, agroforestry and sustainable cultivar production and receives visitors. The preservation of these areas has allowed the maintenance of biodiversity, and therefore new scientific findings. This illustrates the need to plan and develop conservation strategies in the face of global climate change and other anthropogenic threats.

## Supporting information

S1 Table Specimens’ vouchers and locality, and Genbank accession numbers. The specimens obtained in the present study are in bold.
(DOCX)

S1 Fig Consensus phylogenetic tree of genus *Tulostoma*, obtained by Maximum Likelihood inference with ITS, nucLSU and Tef1-α. Numbers on nodes are bootstrap support values.
(PDF)

S2 Fig Heat map for pairwise genetic distance between species. Shorter distances are indicated by darker colours.
(PDF)

S1 Data Genetic p-distances values calculated between species and between reticulated-spore group and other *Tulostoma* species. (XLSX)

S2 Data bPTP results with MOTUs and support values. Sheet 1 is results from simple heuristic search (bayesian) and sheet 2 results from maximum likelihood.
(XLSX)
