# Supplementary material for: Diversity of Neotropical stalked-puffball: Two new species of Tulostoma with reticulated spores
Source: PLoS One. 2023 Dec 13;18(12):e0294672. doi: 10.1371/journal.pone.0294672 (PMC10718411; doi:10.1371/journal.pone.0294672)
Supplement: S1 Table — The specimens obtained in the present study are in bold. (DOCX) [file pone.0294672.s001.docx]

**Table S1.** Specimens’ vouchers and locality, and Genbank accession numbers. The specimens obtained in the present study are in bold. Herbarium acronyms followed Index Herbariorum (https://sweetgum.nybg.org/science/ih/).

| **Species** | **Voucher** | **Locality** | **Genbank accession number** | | | |
| --- | --- | --- | --- | --- | --- | --- |
|  |  |  | **ITS** | **nucLSU** | **ITS+nucLSU** | **Tef1-α** |
| *T. beccarianum* | Finy 2 (GB) | Hungary | - | - | KU843959 | KU519076 |
| *T. beccarianum* | Beccari (S), holotype | Italy | - | - | KX640979 | - |
| *T. brumale* | Jeppson 6427 (GB) | Sweden | - | - | KU843947 | KU519064 |
| *T. calcareum* | Aarnaes 2010-­‐1 (GB) | Spain | - | - | KU519083 | KU843877 |
| *T. calcareum* | Jeppson 6965 (GB), holotype | Sweden | - | - | KU519086 | KU843881 |
|  |  |  |  |  |  |  |
| *T. calongei* | Jeppson 8773 (GB), holotype | Spain | - | - | KU518973 | KU844000 |
| *T. calongei* | Álvarez (AH 13718) | Spain | - | - | KC333067 | - |
| *T.* aff. *cretaceum* | Jeppson 9304 (GB) | Spain | - | - | KU519000 | - |
| *T.* aff. *cretaceum* | Jeppson 6194 (GB) | Spain | - | - | KU518997 | KU843989 |
| *T. cyclophorum* | Gonzáles (AH 16885, GB) | Spain | - | - | KU518983 | KU843961 |
| *T. cyclophorum* | Jeppson 8862 (GB) | Hungary | - | - | KU518985 | KU843963 |
| *T. domingueziae* | MLHC200 | Argentina, Córdoba | HQ667594 | HQ667597 | - | - |
| *T. eckbladii* | Jonsell (TRH 9565) | Norway | - | - | KU519069 | KU843952 |
| *T. eckbladii* | Jørstad (O F-­58850), holotype | Norway | - | - | KU519068 | KU843951 |
| ***T.*** **aff. *exasperatum*** | **UFRN-Fungos 1908** | **Brazil, Piauí** | **OQ672312** | **-** | **-** | **-** |
| *T. exasperatum* | SDBR-CMUNK1819 | Thailand | OQ165118 | OQ159058 | - | OQ172031 |
| *T. exasperatum* | SDBR-CMUNK1815 | Thailand | OQ158993 | OQ159021 | - | OQ172030 |
| *T. excentricum* | Lloyd 15424 (BPI), holotype | USA, New Mexico | - | - | KU519055 | - |
| *T. fimbriatum* | Månsson 991010 (GB), epitype | Sweden | - | - | KU518963 | KU843904 |
| *T. fimbriatum* | Jeppson 5795 (GB) | Sweden | - | - | KU518961 | KU843903 |
| *T. fimbriatum* | Webber (NY), lectotype *T. campestre* | USA, Nebraska | - | - | KC333075 | - |
| *T. fulvellum* | Kabát 970428 (BRA) | Slovakia | - | - | KU518991 | KU844001 |
| *T. giovanellae* | Bartolomé & Alvarez (AH 11641 -­‐ GB) | Spain | - | - | KU519072 | KU843955 |
| *T. grandisporum* | Jeppson 8907 (GB), holotype | Hungary | - | - | KU519003 | KU843924 |
| *T. kotlabae* | Jeppson 7923 (GB) | Slovakia | - | - | KU519027 | KU843973 |
| *T. kotlabae* | Kotlaba (PRM 704203), holotype | Slovakia | - | - | KX576544 | - |
| *T. lloydii* | Lahti 201210 (GB) | Italy | - | - | KU518990 | KU843965 |
| *T. lusitanicum* | Almeida et al. (LISU-­‐MAG 8), holotype | Portugal | - | - | KX576542 | - |
| *T. lysocephalum* | Long 9639 (BPI), holotype | USA, New Mexico | - | - | KU519034 | - |
| *T. melanocyclum* | Jeppson 8815 (GB) | Hungary | - | - | KU519105 | KU843886 |
| ***T. mucugeense*** | **ALCB 141142, holotype** | **Brazil, Bahia** | **OQ626704** | **OQ626710** | **-** | **-** |
| *T. niveum* | Jeppson 7699 (GB) | Sweden | - | - | KU519079 | KU843933 |
| *T. obesum* | Jeppson 8707 (GB) | Spain | - | - | KU518987 | KU843986 |
| *T. obesum* | Cooke 2715 (NY), isotype | USA, Colorado | - | - | KX576541 | - |
| *T. pannonicum* | Jeppson 7803 (GB) | Hungary | - | - | KU519011 | KU843996 |
| *T. pannonicum* | Jeppson 7764 (GB), holotype | Hungary | - | - | KU519010 | - |
| ***T. paratyense*** | **RB 845594, holotype** | **Brazil, Rio de Janeiro** | **OQ626707** | **OQ626712** | **-** | **OQ658839** |
| *T. pseudopulchellum* | Illana, Moreno, Altés (AH 11603), paratype | Spain | - | - | KU519012 | KU843997 |
| *T. pulchellum* | Moravec 132/51 (UPS), paratype *T. hollosii* | Czech Republic | - | - | KX513825 | - |
| *T. punctatum* | Bethel 21 (NY), holotype *T. subfuscum* | USA, Colorado | - | - | KC333074 | - |
| *T. punctatum* | Jeppson 7472 (GB) | Slovakia | - | - | KU518952 | KU843875 |
| ***T. ridleyi*** | **MA Fungi 83796, gleba** | **Singapore** | **OQ626705** | **OQ626711** | **-** | **-** |
| ***T. ridleyi*** | **MA Fungi 83796, exoperidium** | **Singapore** | **OQ626706** | **-** | **-** | **-** |
| *T. rufum* | Lloyd 15542 (BPI), holotype | USA, Alabama | - | - | KU519107 | - |
| *T. simulans* | Jeppson 3844 (GB) | Hungary | - | - | KU519052 | KU843941 |
| *T. simulans* | Jeppson 7865 (GB) | Austria | - | - | KU519049 | KU843938 |
| *T. simulans* | Moravec (PRM 667204), isotype *T. moravecii* | Czech Republic | - | - | KX576546 | - |
| *T. submembranaceum* | Ayala et al. (AH 15132), holotype | Mexico | - | - | KX513826 | - |
| *T. subsquamosum* | Jeppson 9305 (GB) | Spain | - | - | KU519094 | KU843897 |
| *T. subsquamosum* | Jeppson 4956 (GB) | Hungary | - | KU519095 | - | - |
| *T. winterhoffii* | Lode ZfM79/2 (pers. herb. H. Schubert), paratype | Germany | - | - | KU518975 | - |
| *T. winterhoffii* | Jeppson 7761 (GB) | Hungary | - | - | KU518976 | KU843916 |
| *T.* sp. 1 | Jeppson 7762 (GB) | Hungary | - | - | KU518979 | KU843981 |
| *T.* sp. 2 | Jeppson 8701 (GB) | Spain | - | - | KU518981 | KU843983 |
| *T.* sp. 3 | Jeppson 4935 (GB) | Hungary | - | - | KU518978 | KU843918 |
| *T.* sp. 4 | Finy 12 (GB) | Hungary | - | - | KU518960 | KU843902 |
| *T.* sp. 5 | Brůžek 131207 (GB) | Slovakia | - | - | KU519041 | KU843931 |
| *T.* sp. 6 | Jeppson 5996 (GB) | Hungary | - | - | KU519016 | KU843966 |
| *T.* sp. 7 | Finy 1 (GB) | Hungary | - | - | KU519017 | KU843967 |
| *T.* sp. 8 | Jeppson 7795 (GB) | Hungary | - | - | KU519020 | KU843970 |
| *T.* sp. 9 | Jeppson 4966 (GB) | Hungary | - | - | KU519036 | KU843968 |
| *T.* sp. 9 | Jeppson 4976 (GB) | Hungary | - | - | KU519037 | KU843969 |
| *T.* sp. 10 | Jeppson 6198 (GB) | Spain | - | - | KU519030 | - |
| *T.* sp. 10 | Jeppson 3813 (GB) | Hungary | - | - | KU519029 | - |
| *T.* sp. 11 | Jeppson 881114 (GB) | Spain | - | - | KU519031 | KU843978 |
| *T.* sp. 12 | Vidal (AH 15040 -­‐ GB) | Spain | - | - | KU519032 | KU843979 |
| *T.* sp. 14 | Jeppson 5011 (GB) | Spain | - | - | KU519038 | KU843998 |
| *T.* sp. 14 | Jeppson 5004 (GB) | Spain | - | - | KU519039 | KU843999 |
| *T.* sp. 15 | Jeppson 9296 (GB) | Spain | - | - | KU519014 | KU843984 |
| *T.* sp. 16 | Knudsen 99.337 (C) | Russia | - | - | KU519007 | KU843980 |
| *T.* sp. 17 | Equipo mycología UAH (AH 13674, GB) | Spain | - | - | KU519065 | - |
| *T.* sp. 18 | Jeppson 9046 (GB) | Spain | - | - | KU519066 | KU843956 |
| *T.* sp. 19 | Molia 140115-­‐2 (GB) | Cyprus | - | - | KU519077 | KU843957 |
| *T.* sp. 20 | Jeppson 5015 (GB) | Spain | - | - | KU519067 | KU843950 |
| *T.* sp. 21 | Moreno & Altés (AH 11698) | Spain | - | - | KX640986 | - |
| *T.* sp. 22 | MLHC200 | Argentina, Cordoba | HQ667595 | HQ667598 | - | - |
| *Calvatia caatinguensis* | UFRN-Fungos 2945 | Brazil | MG871364 | NG_071245 | - | - |
| *Lycoperdon subperlatum* | KA12-0281 | South Korea | KP340201 | - | - | - |
