# Supplementary figures and images for: Diversity of Neotropical stalked-puffball: Two new species of Tulostoma with reticulated spores
Source: PLoS One. 2023 Dec 13;18(12):e0294672. doi: 10.1371/journal.pone.0294672 (PMC10718411; doi:10.1371/journal.pone.0294672)

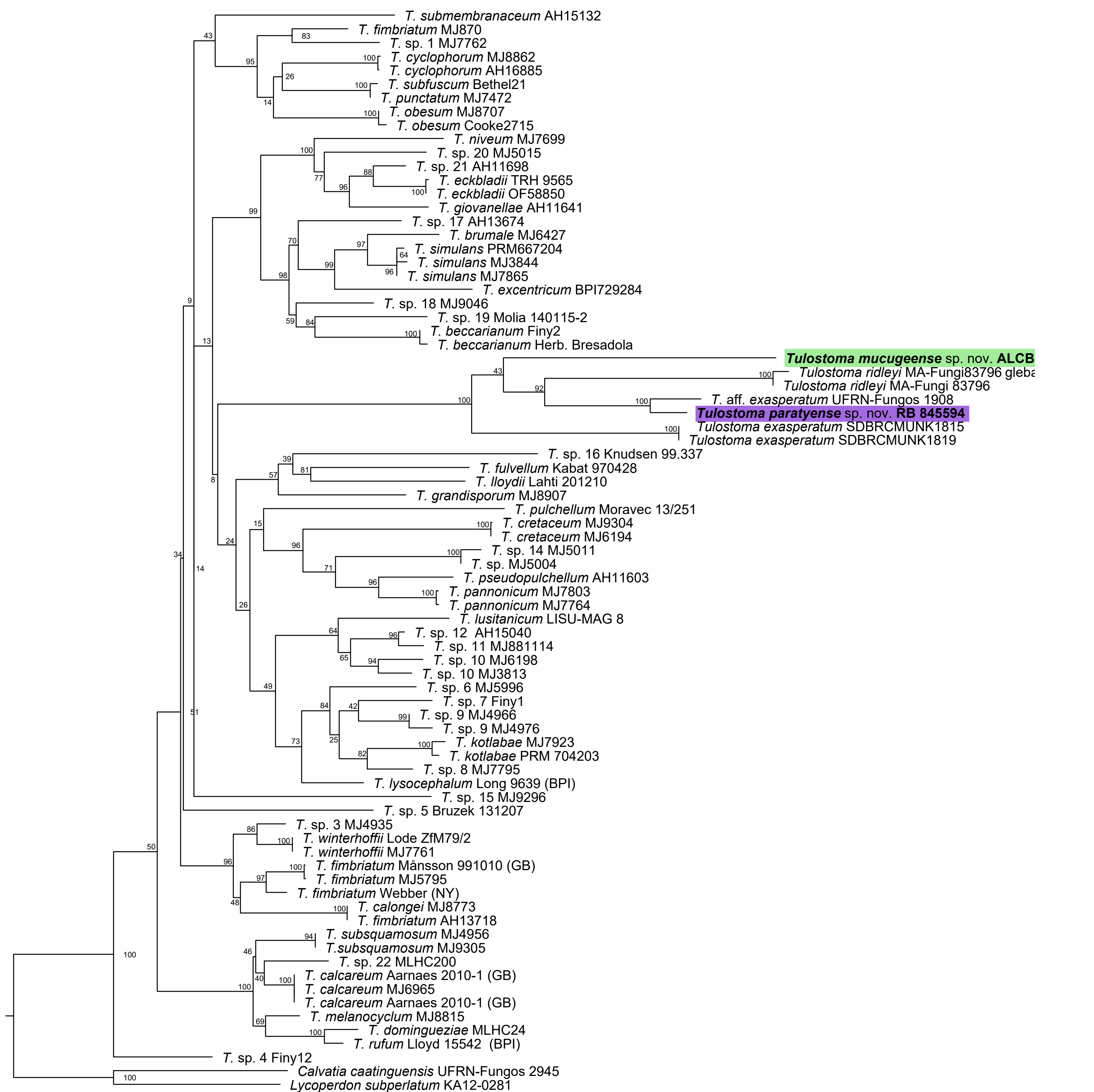

0.05

Supplement: S1 Fig — Numbers on nodes are bootstrap support values. (PDF) [file pone.0294672.s002.pdf]
