# Supplementary material for: Diversity of Neotropical stalked-puffball: Two new species of Tulostoma with reticulated spores
Source: PLoS One. 2023 Dec 13;18(12):e0294672. doi: 10.1371/journal.pone.0294672 (PMC10718411; doi:10.1371/journal.pone.0294672)
Supplement: S2 Fig — Shorter distances are indicated by darker colours. (PDF) [file pone.0294672.s003.pdf]

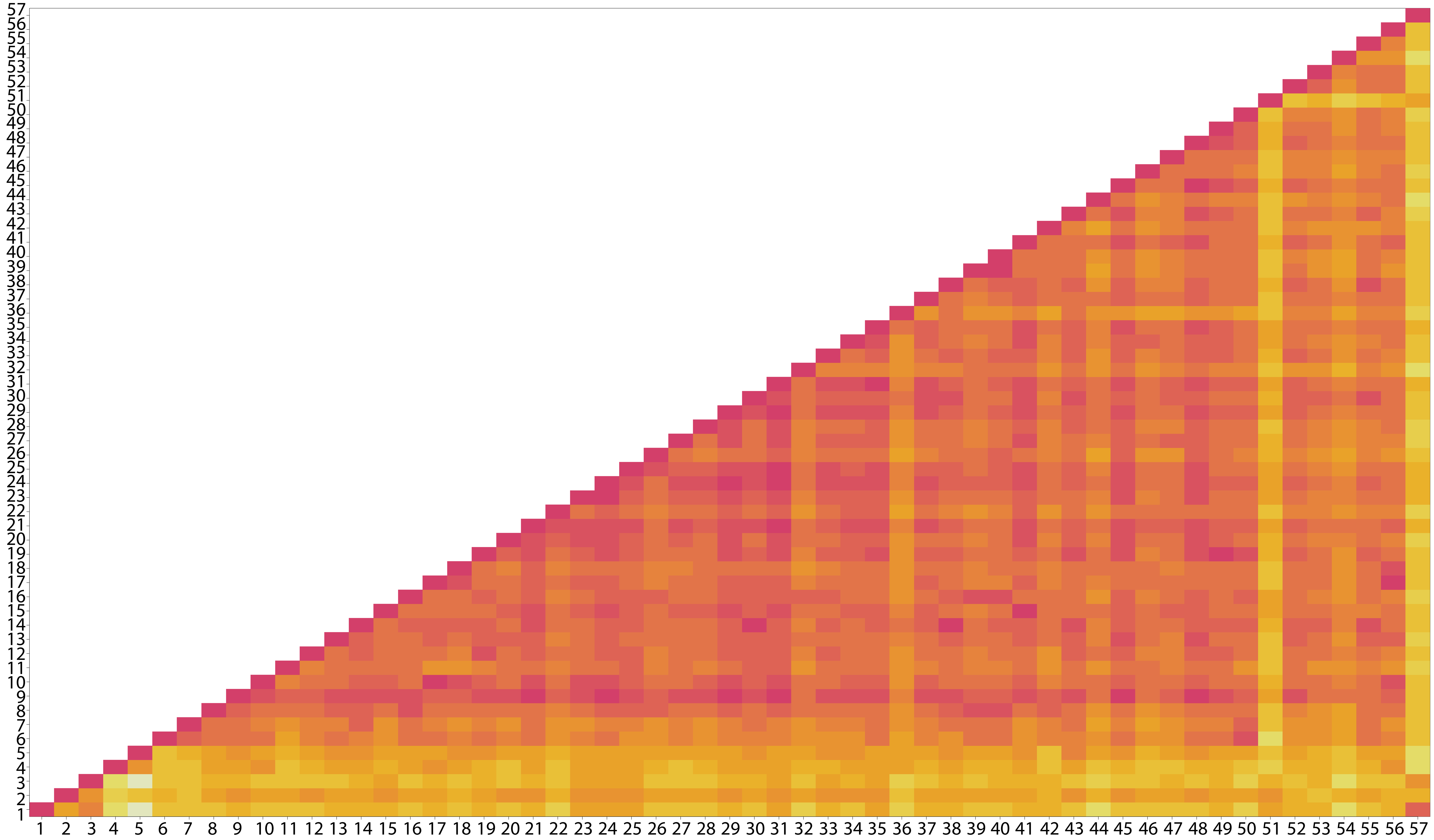

1. Tulstoma paratyense RB 845594
2. T. mucugeense ALCB 141142
3. T. ridleyi MAFungi83796
4. Calvatia caatinguensis UFRNFungos2945
5. Lycoperdon subperlatum KA120918
6. T. beccarianum_Finy2
7. T. beccarianum_Molia2014
8. T. brumale_MJ6427
9. T. caespitosum_AH15040
10. T. calongei_MJ8773
11. T. cretaceum_MJ9304
12. T. cyclophorum_AH16885
13. T. dominguezíae_MLHC210
14. T. dominguezíae_MLHC24
15. T. eckbladii_Sivertsen930717
16. T. excentricum_BPI729284
17. T. fimbriatum_Manson991010
18. T. fimbriatum_MJ4935
19. T. fimbriatum_MJ870
20. T. fulvellum_Kabat970428
21. T. giovanellae_AH11641
22. T. grandisporum_MJ8907
23. T. kotlabae_Finy1
24. T. kotlabae_MJ5996
25. T. kotlabae_MJ7795
26. T. kotlabae_MJ7923
27. T. lloydii_Lahti201210
28. T. lusitanicum_LISUMGA8
29. T. lysocephalum_BPI749235
30. T. melanocyclum_MJ8815
31. T. nanum_MJ4966
32. T. niveum_MJ7699
33. T. obesum_MJ8707
34. T. pannonicum_MJ7803
35. T. pseudopulchellum_AH11603
36. T. pulchellum_Moravec13251
37. T. pygmaeum_Bruzek131207
38. T. rufum_BPI704578
39. T. simulans_MJ3844
40. T. simulans_MJ7865
41. T. sp_AH11698
42. T. sp_AH13674
43. T. sp_Finy12
44. T. sp_Knudsen99_337
45. T. sp_MJ3813
46. T. sp_MJ5011
47. T. sp_MJ5015
48. T. sp_MJ6198
49. T. sp_MJ7762
50. T. sp_MJ9046
51. T. exasperatum SDBR-CMUNK1819
52. T. subfuscum_Bethel21
53. T. submembranaceum_AH15132
54. T. submembranaceum_MJ9296
55. T. subsquamosum_MJ9305
56. T. winterhoffii_ZFM74_2
57. T. aff. exasperatum UFRN-Fungos 1908
